# Transcriptomic Response to Neuromuscular Electrical Stimulation in Muscle, Brain, and Plasma EVs in WT and Klotho-Deficient Mice

**DOI:** 10.3390/ijms26167849

**Published:** 2025-08-14

**Authors:** Catherine Anne Cavanaugh, Amanda E. Moore, Nicholas Francis Fitz, Iliya Lefterov, Radosveta Koldamova

**Affiliations:** Department of Environmental and Occupational Health, University of Pittsburgh, Pittsburgh, PA 15261, USA; cac355@pitt.edu (C.A.C.); aem284@pitt.edu (A.E.M.); nffitz@pitt.edu (N.F.F.); iliyal@pitt.edu (I.L.)

**Keywords:** NMES, extracellular vesicles, Klotho, muscle, brain, RNA-seq, non-coding RNAs, metabolism

## Abstract

Neuromuscular electrical stimulation (NMES) has been shown to improve motor activities and daily living. Prior studies indicated extracellular vesicles (EVs) play a role in cellular communication. Here, we evaluated transcriptomic profiles of tibialis muscle, brain, and plasma-derived EVs following NMES in wild type (WT) and *Klotho* heterozygous (Kl^HET^) mice. Muscle RNA-seq data demonstrated that, in both genotypes, the most upregulated functional categories were related to glucose metabolism and response to insulin, with pathways uniquely affected in each genotype. There was a similarity of the non-coding RNA transcriptome of plasma EVs, with functional patterns suggesting response to oxygen and insulin and long-term synaptic potentiation. The brain transcriptome showed little functional overlap between WT and Kl^HET^ mice. In WT, brain upregulation of genes was related to blood flow and cell adhesion processes, while Kl^HET^ showed upregulation of immune function. The results indicate that similar metabolic function is impacted in the location of stimulation, but the distal impact of stimulation on the brain is associated with Klotho deficiency.

## 1. Introduction

**Figure 1:**
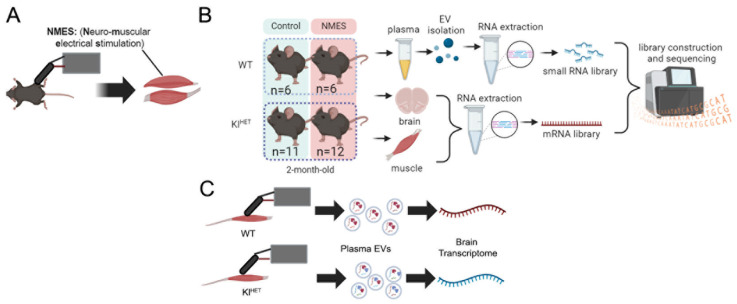
**Study design.** (**A**) Two-month-old WT and Kl^HET^ mice were subjected to NMES on the tibialis anterior and extensor digitorum longus of each leg for two sets of 10 stimulations every other day for five total sessions. (**B**) Experimental groups were assigned, and tissue was collected. Library generation and sequencing occurred with mRNA libraries from the muscle and brain and small RNA libraries from plasma-derived extracellular vesicles (EVs). (**C**) The pictorial experimental results summary indicates similarity in transcriptomic response in the area of stimulation and in EVs, with the results of the brain transcriptome varying. (N: WT 6/group; Kl^HET^ control = 11; Kl^HET^ NMES = 12.) Figures were made using BioRender.com (web-based; accessed 7 August 2025).

Neuromuscular electrical stimulation (NMES) of muscle contractile activity is a noninvasive approach aimed at improving activities of daily living and functional motor ability in elderly and post-stroke patients (Figure 1A) [1,2]. Beneficial for muscular angiogenesis, muscular tetanic specific force, cognitive function, and increasing glycemic control, NMES serves as a supplement to therapeutic recovery efforts and a treatment option with benefit in individuals with metabolic syndrome and venous disease [1,3,4,5]. Given that progressive neurodegenerative diseases such as Alzheimer’s Disease show strong links to inactivity, NMES’s positive effect on blood flow may provide insight into slowing neurodegenerative processes [6].

Emerging information regarding communication between the muscle and brain shows that proteins produced by differentially expressed genes (DEGs), likely carried from the stimulation source by extracellular vesicles (EVs), travel through the bloodstream to the destination tissue, allowing for tissue crosstalk [7]. This finds additional support in similar DEGs from muscle tissue post-direct electrical stimulation and post-aerobic exercise, indicating similar localized benefits and genes with functions such as cell migration, regulating apoptosis, and cell attachment are downregulated in muscle cases of NMES and aerobic exercise compared to controls, while glucose metabolism is upregulated [8]. Further studies indicate that messenger RNAs (mRNAs) and non-coding RNAs (ncRNA) carried by EVs can regulate functions within the tissue, leading to physiological benefits post-exercise [9,10]. Transcriptomic effects are seen by functional RNA molecules that regulate gene expression but do not code for proteins; they are called non-coding RNAs (ncRNAs) and include microRNAs (miRNAs), small nucleolar RNAs (snoRNAs), circular RNAs (circRNAs), transfer RNAs (tRNAs), and small nuclear RNAs (snRNAs), among others [11]. Altered ncRNA expression has been shown to impact synaptic plasticity, neuronal function, and clearing of amyloid-beta and tau, directly linking it to neurodegenerative disease function [12,13]. Therefore, in two of our previous studies, we utilized heterochronic blood exchange and tail vein EV injections to better understand the role of circulating neuroprotective young EVs in improving cognitive function [14,15]. Considering the importance of the EVs’ contents—proteins, lipids, ncRNA, and mRNA—being transported between tissues, greater understanding of how DEGs post-exercise regulate the vesicle contents is an important research question. Exosomes and EVs were shown before to enter the brain by crossing the blood–brain barrier (BBB) or cerebrospinal fluid (CSF) barrier [16], and several mechanisms of BBB penetration, such as protein-mediated transport, receptor-mediated transcytosis, transcellular diffusion, and adsorptive-mediated diffusion, have been noted [17,18].

Exerkines are a class of signaling molecules stimulated by exercise that activate the autocrine, paracrine, and/or endocrine systems [19]. The *Klotho* gene encodes the KLOTHO protein, which is expressed in skeletal muscle, kidney, and the choroid plexus, as well as becoming soluble in plasma when cleaved from the membrane via beta-site amyloid precursor protein cleaving enzyme 1 (BACE1), a disintegrin and metalloproteinase domain-containing protein 10 (ADAM-10) or a disintegrin and metalloproteinase domain-containing protein 17 (ADAM-17) [20]. Because *Klotho*-deficient mouse models display reduced lifespan and cognition, while *Klotho*-overexpressing mice live longer, *Klotho* is a focus of various fields of research, including neurodegenerative, musculoskeletal, and aging research [21,22,23]. Various articles have discussed the involvement of Klotho overexpression in mediating amyloid-beta (Aβ) clearance and improving cognition in AD mouse models, and higher levels of circulating KLOTHO in humans are associated with a lower risk of AD [24]. Therefore, the transcriptomic mimicking of neurodegenerative aging effects provided by the deficiency of KLOTHO is why we use this model [24].

This study, with design indicated in Figure 1, evaluated how NMES treatment affected the muscle and brain transcriptome, as well as non-coding RNA expression in plasma EVs. To determine the role of KLOTHO deficiency on these parameters, we used previously established NMES protocols to compare the NMES effect in wild-type (WT) and KLOTHO-deficient (Kl^HET^) mice [25]. We performed bulk RNA-seq on brain and muscle tissue to determine the effect of treatment and genotype. We showed that in muscle mRNA and plasma-derived EV ncRNA, both genotypes included functional overlap related to insulin response. However, the brain transcriptome showed little functional overlap between WT and Kl^HET^ mice, with WT demonstrating upregulation of genes related to blood flow and cell adhesion processes, while Kl^HET^ showed upregulation of immune function. This study aims to elucidate the role of localized stimulation and metabolic impact on the brain via EV ncRNA trafficking.

## 2. Results

### 2.1. NMES Treatment Activates Metabolic Response in Muscle Tissue Similarly in WT and Kl^HET^ Mice

**Figure 2:**
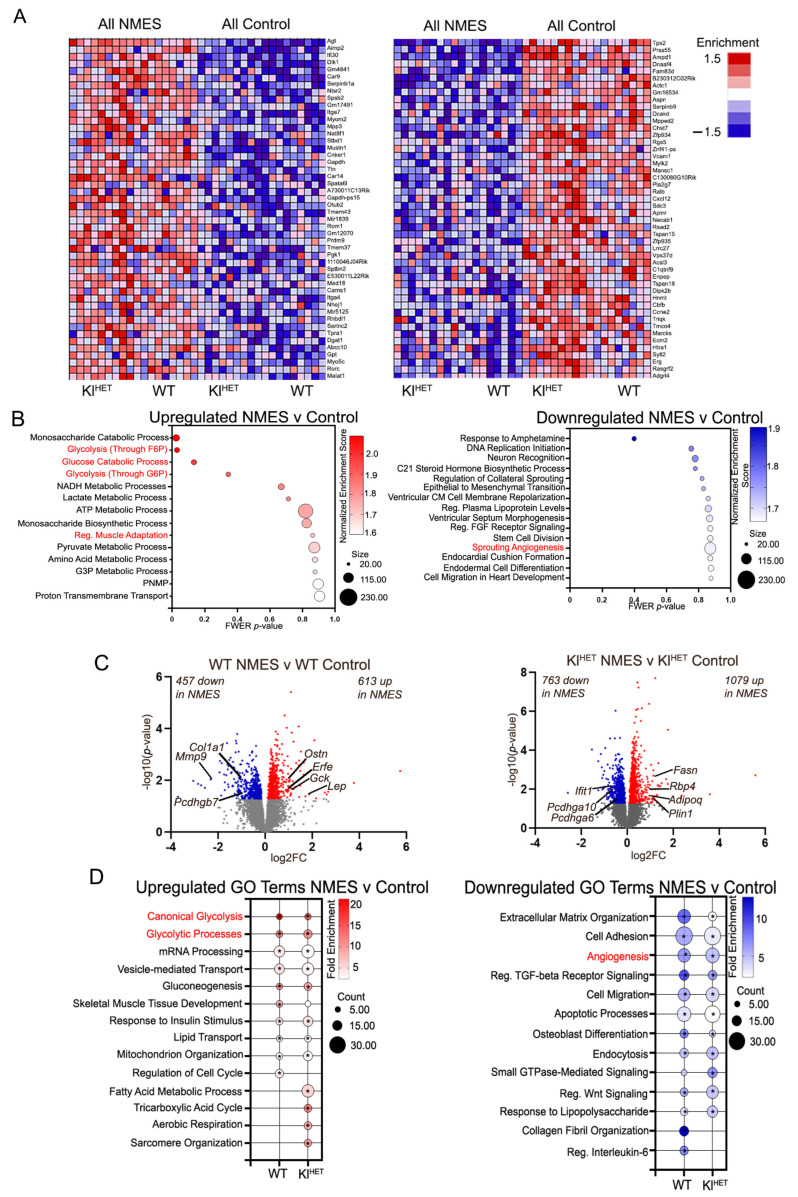
**NMES treatment activates metabolic response in muscle tissue similarly in WT and Kl^HET^ mice**. Gene expression profiling was performed by RNA-seq on muscle dissected from WT and Kl^HET^ mice that underwent NMES treatment, and edgeR was utilized to identify differentially expressed genes between treatment groups. Gene Set Enrichment Analysis (GSEA) was applied to identify top upregulated categories in all NMES vs. all control comparison. (**A**) The heatmap was generated by GSEA and depicts the top up- and downregulated genes from all NMES vs. all control. (**B**) Bubble plots depicting upregulated (**left**) and downregulated (**right**) biological processes from genes in (**A**). (**C**) Volcano plots of DEGs between NMES and controls in WT (**left**) and Kl^HET^ (**right**) at a cut-off of *p* < 0.05. Red denotes significantly upregulated genes, blue denotes significantly downregulated genes, and gray denotes non-significance. (**D**) Bubble plots depicting Gene Ontology (GO) terms for genes from (**C**) with the highest fold enrichment for biological functions, generated using DAVID. Upregulated genes are shown in the right panel and downregulated genes in the left panel. Shown in red font are GO terms overlapping between WT and Kl^HET^ and with GO terms in (**B**). (N: WT 6/group; Kl^HET^ control = 11; Kl^HET^ NMES = 12), * *p* < 0.05.

Muscle gene expression profiling was performed by RNA-seq on dissected muscle from WT and Kl^HET^ mice that underwent NMES treatment, and edgeR was utilized to identify DEGs between treatment groups. Our first goal was to determine the effect of NMES, and we compared muscle-derived mRNA data from all NMES-treated mice to control mice (Figure 2A,B and Supplemental Table S1). The heatmap shown in panel A was generated by the GSEA enrichment scores of differentially expressed genes (DEGs) and demonstrates that top up- and downregulated genes from both genotypes have the same pattern and are affected in the same direction. Some notable genes included are *Agt*, *Dlk1*, *Mustn1*, and *Mylk2*, all of which have previously been related to muscular training and metabolic functions [26,27,28,29]. The top biological processes upregulated by NMES are monosaccharide catabolic processes, glycolysis (through F6P), glucose catabolic processes, and glycolysis (through G6P), regulation of muscle adaptation, G3P metabolic processes, purine nucleoside metabolic processes (abbreviated as PNMP), and proton transmembrane transport, as seen in Figure 2B (left panel). Figure 2B (right panel) illustrates downregulated functions in NMES versus controls and includes the epithelial-to-mesenchymal transition, regulation of plasma lipoprotein levels, regulation of FGF receptor signaling, and sprouting angiogenesis. Terms written in red indicate functional overlap with comparisons in Figure 2B,D. Enrichment reports for selected terms, including the glycolytic process through F6P and the glucose catabolic process, are shown in Supplemental Figure S1. Enrichment plots show a similarity of shape and enrichment score for all NMES vs. all control (Figure S1A), WT NMES vs. WT control (Figure S1B), and Kl^HET^ NMES vs. Kl^HET^ control (Figure S1C), indicating that the biological processes derived from including all genotypes in the NMES vs. control comparison are methodologically sound.

We next compared how each genotype responds to the treatment. The left panel volcano plot in Figure 2C illustrates DEGs in WT NMES versus WT control and the right DEGs from the same comparison in Kl^HET^ mice (Supplemental Table S1). A comparison of the overlap between these gene sets is included in Supplemental Figure S2, with 191 upregulated and 81 downregulated genes overlapping (Supplemental Figure S2A). As shown in Supplemental Figure S2, the top upregulated categories confirmed the GSEA results, namely that NMES increased glucose metabolism. Then, the DEGs from Figure 2C were analyzed using DAVID to compare whether WT and Kl^HET^ experience different responses to NMES treatment. The top upregulated GO term categories in both genotypes include glycolysis, mRNA processing, gluconeogenesis, skeletal muscle tissue development, and response to insulin stimulus (Figure 2D, left). It should be noted that in addition to the common DEGs, the top GO categories also included unique significant genes in each genotype. For example, *Erfe*/erythroferrone, which is highly expressed in skeletal muscle and is a part of gluconeogenesis, was differentially upregulated by NMES only in WT but not in Kl^HET^ mice [30]. Overall, the shared upregulated GO terms between the two genotypes after NMES (glycolysis, gluconeogenesis, and insulin response) are predominantly related to metabolic function, emphasizing the relationship between localized muscular treatment and transcriptomic metabolic benefit. Uniquely upregulated in WT is regulation of cell cycle (*Lep)*. Additionally, *Ostn* (Osteocrin/Musclin), a myokine primarily expressed in skeletal muscle and released during muscle activity, has been shown to enhance physical endurance and is upregulated only in WT but not in Kl^HET^ mice. Uniquely upregulated in Kl^HET^ mice are fatty acid metabolic processes (*Fasn*), tricarboxylic acid cycle (*Sucgl1*), aerobic respiration (*Ndufa8*), and sarcomere organization (*Myom2*).

Notably, the data indicate similar overlap from downregulated DEGs in Figure 2C, left. The top downregulated GO terms that overlap in WT NMES and Kl^HET^ NMES compared to the respective control conditions include extracellular matrix organization, cell adhesion, and collagen fibril organization, with numerous collagen genes such as *Col1a1* and *Col15a1* and metalloproteases such as *Mmp9* (Figure 2D) [31,32]. While cell adhesion is downregulated in both WT and Kl^HET^, the genes comprising the same GO term vary by genotype. Kl^HET^ genes within the category were predominantly protocadherin genes such as *Pcdhga10* and *Pcdhga6,* which are associated with muscle frailty in older adults, while the WT had only three protocadherin genes (*Pcdhgb7*, *Pcdhga4*, and *Pcdh17*) but more collagen genes (*Col1a1*) and integrin genes (*Itga1*) [33]. Notably, the GO term for collagen fibril organization is only downregulated in WT NMES vs. WT control conditions but not in the Kl^HET^ comparison, supporting this gene difference. The comparison of gene lists comprising each GO term provides further support for the conclusion that, while gene function is similar, the specific DEGs comprising each term vary, which indicates a potentially genotype-mediated transcriptomic response to localized exercise.

### 2.2. KLOTHO Deficiency Significantly Affects Muscle Transcriptome

**Figure 3:**
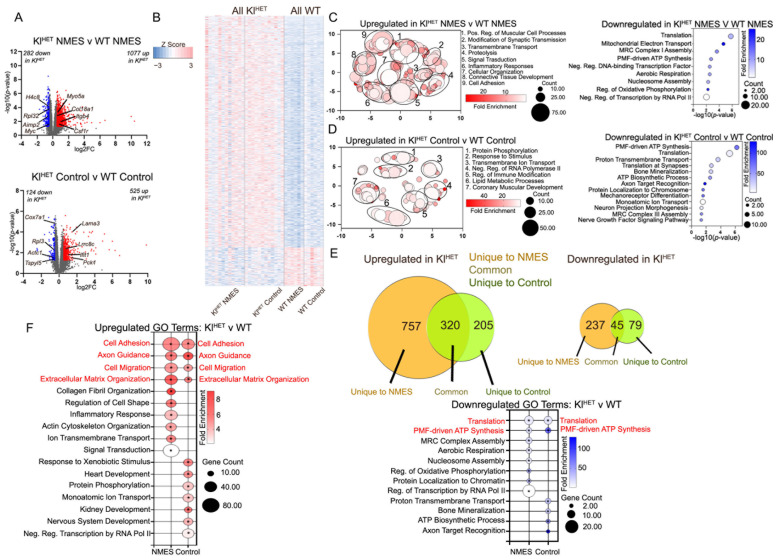
**KLOTHO deficiency significantly affects muscle transcriptome.** Gene expression profiling was performed by RNA-seq on dissected muscle from WT and Kl^HET^ mice that underwent NMES treatment, and edgeR was utilized to identify differentially expressed genes between treatment groups. (**A**) Volcano plots represents differentially expressed genes (DEG) between Kl^HET^ NMES and WT NMES (**upper panel**) and Kl^HET^ control and WT control (**lower panel**) mice at a cut-off of *p* < 0.05 and FC > 0.5. Red denotes significantly upregulated genes, blue denotes significantly downregulated genes, and gray denotes non-significance. (**B**) A heatmap of DEGs. (**C**) REVIGO for upregulated DEGS (**left panel**) and bubble plot for downregulated DEGs (**right panel**) depicting Gene Ontology (GO) terms for biological functions associated with DEGs shown in upper (**A**), generated using DAVID. (**D**) REVIGO for upregulated DEGs (**left panel**) and bubble plot for downregulated DEGs (**right panel**) depicting Gene Ontology (GO) terms for biological functions associated with DEGs shown in lower (**A**), generated using DAVID. (**E**) Venn diagram represents common and unique DEGs in NMES and control mice, as shown in (**A**). Red font indicates GO term overlap. (**F**) Bubble plots depicting GO terms with the highest fold enrichment for biological functions generated using DAVID. Upregulated genes are shown in the right panel and downregulated genes in the left panel. (N: WT 6/group; Kl^HET^ control = 11; Kl^HET^ NMES = 12), * *p* < 0.05.

Comparisons by genotype examined through muscle gene expression profiling, as shown in Figure 2, identified DEGs between genotype groups to indicate the role of klotho deficiency in skeletal muscle (Figure 3A and Supplemental Table S1). The volcano plot in Figure 3A (upper panel) shows 1077 up- and 282 downregulated DEGs when comparing NMES treatment by genotype, including genes such as *Aimp2,* which is likely involved in the pathogenesis of Parkinson’s disease and related to protein synthesis and cell death [34]. Additionally, the lower panel in Figure 3A notes 525 upregulated genes, such as *Lama3,* which is involved in the structural organization of skeletal muscle, and *Pck1*, which is involved in both carbohydrate and lipid metabolism, as well as 124 downregulated DEGs, when comparing control conditions by genotype [35,36]. To determine similarities between gene expression in Kl^HET^ and WT genotypes, we created a heatmap of DEGs from Figure 3A, finding that most top upregulated and downregulated genes are impacted in the same direction for each genotype, regardless of treatment (Figure 3B). Given the large number of upregulated DEGs, REVIGO was used for visualization of GO terms, while bubble plots remained a better representation of GO terms for downregulation. Genotype comparisons within NMES treatment show upregulation of muscular cell processes (*Col18a1*), cellular organization (*Csf1r* and *Itgb4*), and connective tissue development (*Myo5a* and *Col18a1*) and a downregulation of translation (*Aimp2* and *Rpl32*) and transcriptional processes (*Myc*) (Figure 3C). In Figure 3D, control condition comparisons by genotype revealed upregulation in immune modification (*Ifit1*), lipid metabolism (*Pck1* and *Lrrc8c*), and coronary muscular development (*Lrp1*), while translation (*Rpl3*) and transmembrane transport (*Cox7a1*) were downregulated. The increased expression of genes associated with the immune response in Kl^HET^ NMES mice vs. WT NMES suggests that the lack of KLOTHO predisposes the more vulnerable animals to immune dysregulation following exercise, a process which notes additional immune stress [37,38].

The Venn diagram illustrates a larger overlap in common upregulated DEGs than downregulated DEGs (Figure 3E) and provides support for the greater GO term overlap in upregulated terms between the NMES and control conditions than downregulation, as shown in Figure 3F.

Notably, shared upregulated GO terms related to structural function include cell adhesion, cell migration, and extracellular organization, all of which share a substantial percentage of genes when comparing exercise conditions. This is likely due to genotype’s impact on animal genome. However, collagen fibril organization and actin cytoskeleton organization are only upregulated in Kl^HET^ versus WT in the NMES treatment condition, which indicates a potential structural increase in muscle that is a result specific to KLOTHO-deficient mice from NMES exercise treatment (Figure 3F, left). The downregulated GO term overlap is minimal and only includes processes related to translation and proton motive force-driven ATP synthesis (Figure 3F, right).

### 2.3. Functional Patterns Emerge in Small Non-Coding RNA Signatures of Plasma EVs Associated with NMES in WT and Kl^HET^ Mice

**Figure 4:**
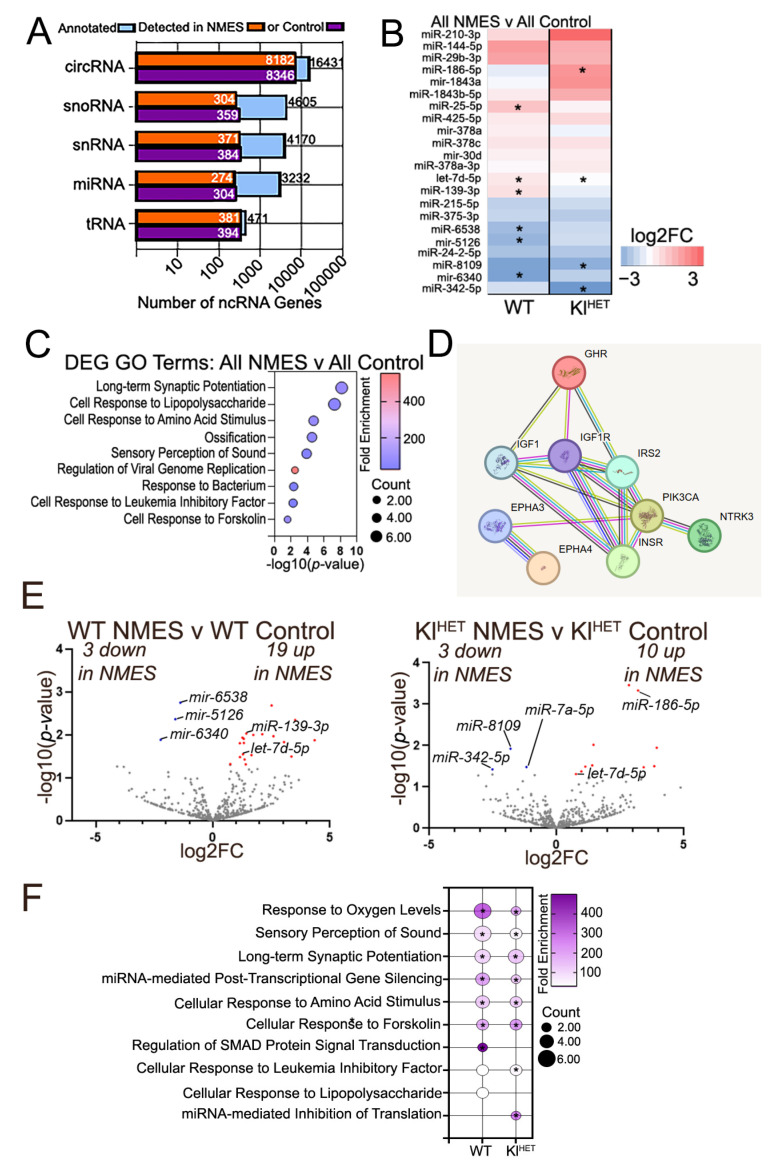
**Functional patterns emerge in RNA signatures of plasma EVs associated with NMES in WT and Kl^HET^ mice.** RNA was isolated from plasma EVs of WT and Kl^HET^ mice after NMES, followed by small non-coding RNA sequencing, to determine changes in EV cargos associated with experimental groups. (**A**) Bar graphs showing the alignment for 5 types of non-coding RNAs detected in EV samples (circRNA, snoRNA, snRNA, miRNA, and tRNA) selected for alignment, with the number of annotations in each treatment group. (**B**) Heat map of fold change in miRNA between all NMES vs. all control demonstrates the similarity of the effect in both genotypes. NMES and control include the respective mice from both genotypes. (**C**) GO terms from DEGs miRNAs shown in (**B**) are visualized. (**D**) STRING interactome for predicted miRNA targeted genes in skeletal muscle GO terms. (**E**) Volcano plots of differentially enriched miRNAs between NMES and control in WT (**left**) and Kl^HET^ (**right**). Red denotes significantly upregulated genes, blue denotes significantly downregulated genes, and gray denotes non-significance. (**F**) GO terms from miRNAs in (**E**) are visualized. (N: WT 6/group; Kl^HET^ 8/group.) * *p* < 0.05.

To better understand how NMES treatment affects plasma EV non-coding transcriptomics, we isolated EVs from plasma after NMES and generated small non-coding RNA-seq libraries (ncRNA). EV characterization indicated successful isolation and no significant size and concentration difference between genotypes nor exercise conditions (Figure S3). Alignment data from both treatments indicate that detected ncRNA readability is similar between NMES and control conditions (Figure 4A and Supplemental Table S2). As with muscle, we first determined the effect of treatment combining both genotypes. The heatmap shown in Figure 4B illustrates differentially enriched miRNA for all NMES versus all control conditions and shows similar directionality between WT and Kl^HET^ comparing all NMES and all control samples. Using the DEG miRNA from Figure 4B, DAVID GO term analysis was conducted, finding that the top regulated processes likely modified by EV contents include long-term synaptic potentiation, cellular response to lipopolysaccharide, and cellular response to amino acid stimulus (Figure 4C). Then, miRNA from Figure 4B was aligned to potential gene matches using miRDB to identify GO term categories of the top regulated processes. A STRING diagram was created utilizing the shared *let-7d* and aligning with the representative GO term “insulin-like receptor signaling” (Figure 4D). Genes such as *Insr* and *Igfr,* which directly modify metabolic and immune function, were noted [39]. We then explored how WT and Kl^HET^ mice reacted individually to the treatment by comparing the NMES group to the control in each genotype. Despite the minimal overlap in miRNAs beyond let-7d-5p, which contributes to inflammatory responses (Figure 4E), a larger pattern of functional overlap emerged in Figure 4F [40]. Figure 4F indicates overlap in functional categories associated with differentially enriched miRNAs, such as response to oxygen levels and long-term synaptic potentiation. Supplemental analysis of circRNA plasma signatures is included in Supplemental Figure S4.

### 2.4. Effect of NMES on Brain Transcriptome Indicates Little Overlap in WT Compared to KlHET

**Figure 5:**
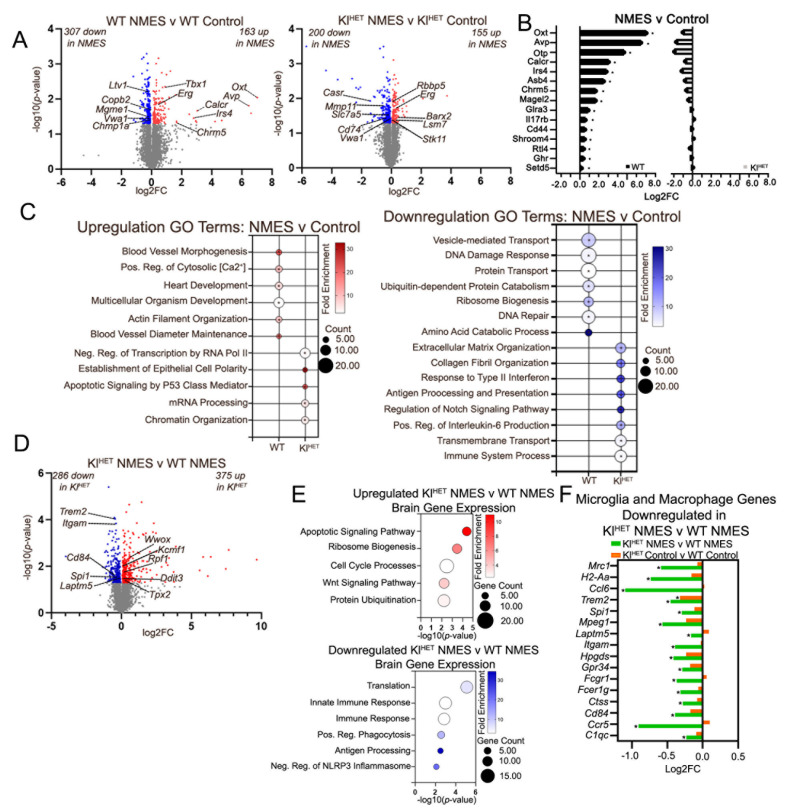
**Effect of NMES on brain transcriptome indicates little overlap in WT compared to Kl^HET^.** Gene expression profiling was performed by RNA-seq on dissected brain from WT and Kl^HET^ mice that underwent NMES treatment, and edgeR was utilized to identify differentially expressed genes between treatment groups. (**A**) Volcano plots represent differentially expressed genes (DEGs) between NMES and control in WT mice (**left panel**) and in Kl^HET^ (**right panel**) at *p* < 0.05. Red denotes significantly upregulated genes, blue denotes significantly downregulated genes, and gray denotes non-significance. (**B**) Bar graph represents selected DEGs from WT-NMES vs. WT-control in both genotypes. (**C**) The bubble plot denotes top GO terms with the highest fold enrichment in each genotype from both upregulated (**left**) and downregulated (**right**) DEGs as generated by DAVID. (**D**) Volcano plots represent differentially expressed genes (DEGs) between Kl^HET^ NMES and WT NMES at *p* < 0.05. Red denotes significantly upregulated genes, blue denotes significantly downregulated genes, and gray denotes non-significance (**E**) Bubble plots depicting Gene Ontology (GO) terms for biological functions associated with DEGs shown in panel (**D**), generated using DAVID. Upregulated terms are shown in the upper panel and downregulated terms in the lower panel. (**F**) Stacked bar graph represents a comparison of fold change in selected microglia and macrophage DEGs downregulated in Kl^HET^ NMES vs. WT NMES. (N: WT 6/group; Kl^HET^ control = 11; Kl^HET^ NMES = 12.) * *p* < 0.05.

To evaluate whether NMES treatment affected the brain transcriptome, we performed bulk RNA-seq on the frontal cortex from WT and Kl^HET^ animals and used edgeR to identify DEGs between treatment groups (Figure 5A and Supplemental Table S1). Volcano plots for WT NMES versus WT control in the left panel of Figure 5A and Kl^HET^ NMES versus Kl^HET^ control in the right panel of Figure 4A exhibit the differences in DEGs after NMES treatment (Figure 5A). In WT mice, among the top DEGs upregulated by the treatment were *Oxt*, *Avp*, *Erg*, *Chrm5*, and *Irs4*, which are involved in metabolic function, blood flow, and synaptic plasticity [41,42,43] (Figure 5B left panel). As shown in Figure 5B, these genes were significantly upregulated in WT-NMES vs. control (black bars) but not in Kl^HET^ NMES vs. their control (gray bars on the right), suggesting a genotype-dependent response to NMES. Using a dot plot to represent DAVID GO terms from DEGs in Figure 5A, we discovered no overlap between biological processes associated with the DEG in both genotypes after NMES (Figure 5C). NMES treatment in the WT group indicated upregulated GO terms, including blood vessel morphogenesis (*Edn1*), blood vessel diameter maintenance (*Avp*), and positive regulation of cytosolic calcium ion (*Oxt* and *Calcr*), while Kl^HET^ mice displayed establishment of epithelial cell polarity (*Cyth1*), apoptotic signaling by p53 mediator (*Wwox*), and mRNA processing (*Barx2*, *Lsm7*, and *Stk11*) (Figure 5C, left panel). Downregulated GO terms from WT mice include vesicle-mediated transport (*Copb2* and *Chmp1a*), DNA damage response (*Mgme1*), and ribosome biogenesis (*Ltv1*), while the Kl^HET^ comparison includes extracellular matrix organization (*Vwa1*), collagen fibril organization (*Col3a1*), and positive regulation of interleukin-6 product (*Cd74*); the neurological pathway response to treatment may be genotype-specific (Figure 5C, right panel). Interestingly, the only gene which was upregulated by NMES in both genotypes is *Erg*, a transcription factor that impacts endothelial junction stability and angiogenesis [44]. The only shared downregulated gene is *Vwa1,* which is involved in extracellular matrix organization and the mutation of which results in delayed neuromuscular responses and impaired motor coordination [45,46].

To evaluate the interaction between genotype and exercise condition in the brain, we evaluated DEGs from Kl^HET^ NMES versus WT NMES, with DEGs shown on the volcano plot in Figure 5D. The 375 genes upregulated in Kl^HET^ NMES versus WT NMES were represented by the GO terms apoptotic signaling pathway (*Wwox*), ribosome biogenesis (*Rpf1*), cell cycle processes (*Cdc73* and *Ddit3*), Wnt signaling pathway (*Wwox*, *Cdc73*, and *Ddit3*), and protein ubiquitination (*Kcmf1*) (Figure 5E, upper panel). Downregulated GO terms included translation (*Rpl3*), innate immune response (*Trem2* and *Cd84*), immune response (*Ccr5*), positive regulation of phagocytosis (*Fcrg1*, *Itgam*, and *Fcerg1*), and negative regulation of NLRP3 inflammasomes (*Trem2*). Notably, GO terms downregulated in Kl^HET^ NMES versus WT NMES are predominantly related to immune function, associated with macrophages and microglia. We used published scRNA-seq data to identify microglia- and macrophage-specific DEGs and compared the fold change (NMES vs. control) in WT and Kl^HET^ (Figure 5F) [47,48]. The only significant gene in both NMES and control comparisons is *Trem2*, the deficiency of which was shown to diminish microglia barrier and amyloid plaque growth in AD mouse models [49].

## 3. Discussion

The purpose of this experiment was to elucidate the transcriptomic impact that localized NMES treatment has on brain, muscle, and plasma EV signatures. Within the experiment, we showed that DEGs within stimulated muscle from NMES are related to metabolic functions and cellular structural modifications. In muscle tissue, genes related to insulin regulation and metabolic modification, such as *Agt*, *Lep,* and *Gck* in WT comparisons and *Adipoq*, *Plin1*, and *Rbp4* in Kl^HET^ comparisons, were differentially expressed for the NMES condition compared to the control, indicating a metabolic pathway response similarity between genotypes in local tissue (Figure 2A,D). These genes, specifically *Lep*/leptin and *Adipoq*/adiponectin, are associated with physical activity and therapy in older adults with sarcopenia [50,51]. For example, these genes are present in several processes, including cellular metabolism, energy balance, and inflammation reduction, with LEP resistance leading to weight gain, cardiac inflammation, and hypertension [52]. The upregulation of *Lep* within our data indicates greater LEP production in direct areas of stimulation, thus proposing a metabolically favorable local effect of NMES treatment, which could play a role in weight maintenance for WT organisms but not Kl^HET^ (Figure 2C, left panel). Evaluation of previous work indicates *Lep*’s predominant role in obesity-related disruption of synaptic plasticity due to its strong link to obesity and its strong relationship to insulin-related genes [53,54]. Therefore, ncRNA analysis from plasma samples indicating contents that regulates trafficking content that targets genes like *Lep,* provides support for the conclusion that localized stimulation may impact the release of EV contents to distal organs (Figure 4D). Therefore, our data supports the conclusion that similar biological results are achieved via predominantly different DEG pathways in localized tissue, which is likely a function of KLOTHO deficiency.

While the brain data indicates a response to NMES, which supports the hypothesis that localized NMES has distal effects, a difference in both DEG function and composition is present. Only WT brain data shows enrichment of several genes related to metabolic function, cognition, and blood flow, including *Oxt*, *Avp*, *Otp*, *Calcr*, *Irs4*, *Asb4*, *Chrm5*, *Magel2*, *Glra3*, *Il17rb*, *Cd44*, *Shroom4*, *Rtl4*, *Ghr*, and *Setd5* [41,55,56]. Notably, results of enriched *OXT* and *AVP* induce both insulin and glucagon for increased metabolic functioning, so seeing a modification in brain data from local stimulation indicates promising neurological metabolic benefits from localized activity [57,58]. Importantly, modified brain CD44 levels were related to lipid metabolic benefits in lipid metabolic processes and insulin resistance, commonly associated with obesity, diabetes, and inflammatory disease [59,60]. Given that *Cd44* is downregulated, the role of insulin resistance is decreased, and lipid metabolism is increased, this indicates that the metabolic pathway effects of NMES are consistent in whole-brain tissue and muscle tissue in WT conditions. Importantly, none of these genes are noted as DEGs for Kl^HET^ NMES animals versus the controls, which see very few genes related to glucose metabolic processes, including *Pomc*, *Stk11*, *Gk*, and *Spop*. While previous works have evaluated the role NMES has in muscle mass, heart rate, bone density, and blood glucose, our recent findings provide specific genetic support explaining how this treatment has widespread metabolic impact, as exhibited by mRNA and ncRNA between KLOTHO-deficient and WT animals, which is a novel finding [61,62].

Notably, the microglia and macrophage gene data indicate significant differences between KLOTHO-deficient and WT treatment conditions related to immune function, which is strongly tied to neurological disease (Figure 5F) [63]. Our previous data comparing the brain of untreated 9-month-old WT and Kl^HET^ mice demonstrated a significant increase in disease-associated microglia genes (*Apoe*, *Trem2*, *Tyrobp*) in Kl^HET^ mice, suggesting neurodegeneration [14]. Given the significance of TREM2 variants in neurodegenerative disease and impaired microglial functioning, our findings indicate that the interaction between NMES and KLOTHO deficiency may be explained by a dysfunction in signaling processes necessary for immune recovery [49]. The significant downregulation of these immune-related genes in Kl^HET^ NMES versus WT NMES but not Kl^HET^ control versus WT control is consistent with previous exercise studies that note short-term exercise-induced immune stress as a means of strengthening the immune system in the long term [64]. Given sample collection less than 24 h following the final round of treatment, the difference between Kl^HET^ and WT animals is likely indicative of the interplay between KLOTHO deficiency and localized exercise treatment on the brain. This result provides support for the argument that NMES differentially metabolically impacts KLOTHO-deficient models in the brain.

GO term analysis of both muscle and brain mRNA indicated genetic modifications for processes involving structural elements like muscle sarcomere organization and blood vessel organization, which indicates metabolic benefits to NMES treatment. Within the muscle data, there is an upregulation of muscle adaptation for all NMES mice, which indicates muscular growth as a result of exercise-induced contractions (Figure 2B) [65]. Specifically, the upregulated genes *MYBPH*, *MYH6*, and *MYOM2,* which regulate myosin binding, indicate that sarcomere re-organization from NMES increases myosin binding to allow for muscle contractions, which increase muscle strength and size [66]. Increased muscular strength related to fast-twitch muscles in the EDL has been reported by previous NMES trials, so our results support a genetic modification mechanism for structural change to achieve increased metabolic action [67]. Additionally, the downregulation of angiogenesis, which is associated with modifications in capillary regression and metabolic diseases like type 2 diabetes, in both WT and Kl^HET^ conditions supports this finding [68,69]. Notably, upon comparing GO terms from each genotype as stratified by treatment condition, similar muscular structural benefits were present by genotype. Both treatment and control conditions for Kl^HET^ saw an upregulation in broad structural modifications, like cell adhesion, axon guidance, cell migration, and extracellular matrix organization; the NMES treatment specifically resulted in collagen fibril organization, regulation of cell shape, and actin cytoskeleton organization (Figure 3F). These results indicate that benefits from NMES treatment likely modify muscle structural components differently under KLOTHO deficiency, which is a novel finding. However, comprehensive histological analysis of muscle tissue was not performed within the scope of this experiment.

Genes related to brain vasculature differences emerged between genotypes upon comparison by treatment. Increased brain vasculature, especially in areas for memory consolidation, as in hippocampal vascularization, has been shown to improve cognitive performance [70]. Therefore, the speculative upregulation of blood vessel morphogenesis and blood vessel diameter maintenance from transcriptomic results in NMES treatment conditions as compared to controls signifies a potential blood flow benefit to WT conditions as a result of localized exercise (Figure 5D). However, Kl^HET^ shows GO term downregulation of collagen fibril organization, and considering its regulatory role in maintaining vascular stiffness and strength, this transcriptomic response could be an indication of vasculature modification via NMES exercise (Figure 5D) [71]. Therefore, NMES likely modifies brain vasculature via differing modalities in WT and Kl^HET^ animals. The structural differences could be explained by the previously established role of KLOTHO deficiency in cranial vascular calcification, necessitating different modalities for blood flow [72,73]. The similarity of certain functions with a difference in gene expression may be partially explained by the shared upregulated gene (*Erg)*. Commonly known as a master transcription factor, *ERG* promotes transcription factor cascades regulating genes associated with blood flow and BBB structural maintenance via endothelial homeostasis [74].

To assess the contents of the transport mechanism between muscle and brain, we analyzed ncRNA signatures from plasma EVs. We chose to focus on miRNA data from this set due to little significance in the other types in response to NMES. However, analysis of circRNA data is included in the Supplemental Results due to its high quantity of EV contents, as demonstrated in Figure 5A, while simultaneously being related to unspecific processes, i.e., phosphorylation, protein phosphorylation, and various protein metabolism and transport functions (Supplemental Figure S4D and Supplemental Table S2). Notably, miRNA significant in all NMES versus all control comparisons show similar directionality in both WT treatment comparisons and Kl^HET^ treatment comparisons (Figure 5B). Biological responses such as response to oxygen levels and long-term synaptic potentiation are shared by treatment between genotypes (Figure 4F). The only shared miRNA upregulated in each genotype group was let-7d-5p, which belongs to the let-7 family of miRNAs, which broadly regulate development [75]. More recent research indicates that let-7d-5p presence regulates insulin signaling targets, correlates to increased obesity, and induces microglial release of inflammatory cytokines [76,77]. In previous human acute muscular exercise trials, the presence of let-7d-5p was upregulated and correlated with inflammatory responses [36]. Functional overlap in EVs parallels that in muscle, providing support for the argument that localized muscular stimulation creates rejuvenation in plasma. However, distal impacts of NMES across the BBB reveal KLOTHO-mediated functional differences. It is important to note that due to their size, EVs are difficult to isolate, and once isolated, EVs vary in contents [78]. While we provide evidence that EVs were successfully isolated from plasma (Figure S3), we did not perform genetic protein tagging for specific EV contents in vivo [79]. Tracking EVs from their release point in the muscle directly into the plasma and directly up to the choroid plexus without mediation from other organ systems would provide definitive proof for our proposed mechanism. However, these techniques are beyond the scope of our study. Additionally, it is notable that peripheral nerves may be stimulated as a result of NMES, which can influence changes in gene expression related to inflammation [80,81]. However, processes modifying peripheral nerve stimulation are also beyond the scope of our study.

Overall, the results of our study indicate that transcriptomic effects of localized muscular stimulation in the brain are affected by KLOTHO deficiency. While sharing similar biological functions in both muscle mRNA and plasma-derived EV ncRNA, the specific genes and ncRNAs responsible for the same biological processes varied by KLOTHO deficiency status. Brain mRNA data showed both differing biological function and gene expression, indicating diverging biological processes as a consequence of communication with the stimulated area. Therefore, the transcriptomic impacts of localized stimulation diverged as a result of KLOTHO deficiency. These diverging data sets may indicate that future klotho-deficient models may require unique treatment paths to slow neurodegeneration.

## 4. Materials and Methods

Mouse Model: Animal studies were approved by the University of Pittsburgh Institutional Animal Care and Use Committee, governed by regulatory standards from the Guide for the Care and Use of Laboratory Animals and the United States Department of Health and Human Services. Wild-type (WT) and KLOTHO HET (Kl^HET^) mice were internally bred for this experiment and randomly assigned to either the control group or the NMES treatment group. The Kl^HET^ mouse strain used for this research project, B6;129S5-*Kl^tm1Lex^*/Mmucd, RRID:MMRRC_011732-UCD, was obtained from the Mutant Mouse Resource and Research Center (MMRRC) at the University of California at Davis, an NIH-funded strain repository, and was donated to the MMRRC by Lexicon Genetics Incorporated (The Woodlands, TX, USA). Mice were housed in a 75 °C, 12:12 h light–dark cycled room with unrestricted access to standard food and water.

NMES: Electrical stimulation was performed with a Neuromuscular Stimulator (Empi 300 PV, Boston, MA, USA) at 9 mA, 150 μs pulse duration, 50 Hz frequency, and 5 s on/10 s off with 0.5 s ramp up/down, under 2.5% isoflurane anesthesia. Mice were shaved on both right and left hind legs along the sciatic nerve, and exposed skin was coated in O.C.T. compound to ensure proper probe placement and to prevent dermal burns. The double-pronged pen attachment was placed at the piriformis, causing leg extension. NMES was delivered in sets of 10 consecutive stimulations per side, oscillating between each leg after each set, for a total of 2 sets on each leg. NMES mice received treatment every other day for a total of 5 treatment sessions. Perfusions and sample collection occurred 24 h after the final treatment session. Control mice received 15 min of 2.5% isoflurane anesthesia exposure each day the NMES treatment group received stimulation.

Sample Collection and Storage: Avertin-anesthetized mice were perfused with chilled PBS after blood was collected intracardially with an EDTA-filled needle. Blood was centrifuged at 13,000× *g* for 5 min for separation of plasma and downstream processing. The grouping of tibialis anterior (TA) and extensor digitorum longus (EDL) muscles was collected from each leg, along with whole-brain tissue. All samples were snap-frozen in dry ice before long-term storage in −80 °C.

Isolation of EVs from Plasma: Plasma samples were thawed at 4 °C and 400 µL was processed with Norgen Biotek’s Plasma/Serum Exosome Purification kit (CAT# 57400, Thorold, ON, Canada) following the manufacturer’s instructions. Aliquots of EV solution were kept for characterization, and the remaining portion of the solution was immediately used for RNA extraction.

Extracellular Vesicle Characterization: All EVs were characterized by Nanosight NTA software (v 3.4) to determine their size and concentration. EV solution was diluted 1:500 and ran through tubing at 0.02 mL/min. EV protein markers were confirmed with Exo-Check Exosome Antibody Array (SBI EXORAY210B-8, Palo Alto, CA, USA) following manufacturer instructions.

RNA Extraction from EVs: Total RNA was isolated from the 190 µL elution of EV solution with Norgen Biotek’s Exosomal RNA Isolation Kit (CAT# 58000), following the manufacturer’s instructions. Quality and concentration were monitored by the RNA Pico 600 Assay Kit for the Agilent 2100 Bioanalyzer System (5067-1513, Waldbronn, Germany).

Isolation of RNA from Brain: A total of 30 mg of prefrontal cortex tissue was subjected to RLT buffer from RNeasy Mini Kit (Qiagen 74106, Hilden, Germany) with 10% beta-mercaptoethanol added and passed through a 25G needle. The homogenate was transferred to QIAshredder (Qiagen 79656, Hilden, Germany) for further dissociation. The remaining manufacturer protocol was followed to complete the RNA isolation. Quality and concentration were analyzed via RNA Nano 600 kit for Agilent 2100 Bioanalyzer (5067-1511, Waldbronn, Germany).

Isolation of RNA from Muscle: Muscle sections weighing 50 mg were powdered by the addition of N_2_ and manually dissociated via mortar and pestle. QIAzol (Qiagen 5,346,994, Hilden, Germany) was added to the powdered muscle and passed through a 25G needle. Homogenate was added to QIAshredder and the RNeasy Mini Kit protocol was followed for the duration of RNA isolation. Quality and concentration were analyzed via RNA Nano 600 kit for Agilent 2100 Bioanalyzer (5067-1511, Waldbronn, Germany).

RNA Library Generation from EVs: Small non-coding libraries were produced by New England BioLabs’ NEBNext Multiplex Small RNA Library Prep Set (E7300S and E7580S, Ipswich, MA, USA). Library quality was assessed on samples by the High Sensitivity DNA Assay Kit for the Agilent 2100 Bioanalyzer System (5067-4626). Samples were sequenced by the Health Sciences Sequencing Core at University of Pittsburgh Children’s Hospital, Pittsburgh, PA, USA, on the NextSeq 2000 machine generating single-end reads. Data was received in .fastq.gz formatting.

RNA Library from Brain and Muscle Tissue: The extracted RNA was sent to Novogene Co. located in Sacramento, CA, USA, for the generation of total RNA libraries and sequencing on a NovaSeq X Plus Series (PE150) machine. Data was received in .fq.gz formatting.

### 4.1. RNA Alignment and Differential Enrichment

Brain and muscle mRNA data were aligned with Rsubread (v 2.16.1), annotated with the org.Mm.eg.db package (v 3.19.1), and analyzed with edgeR (v 4.0.16) [82]. Gene ontology (GO) terms were generated in DAVID with a *p*-value cut-off of <0.05 for Figure 2 and Figure 4 and a *p*-value cut-off of <0.05, followed by a fold-change cut-off of >0.5 for Figure 3 [83,84]. Non-coding RNA library data were aligned and quality-checked by STAR (v 2.5.3a) and analyzed with COMPSRA (v 1.0.3) and DESeq2 (v 1.42.1). Gene ontology (GO) terms were generated in DAVID with a *p*-value cut-off of <0.05. Selected gene targets were analyzed using miRDB at target score cut-off > 50. Representative gene visualization was created utilizing the STRING interaction network database. Using the muscle mRNA data, Gene Set Enrichment Analysis (GSEA) (v.4.3.2) was conducted [85,86].

### 4.2. Statistical Analysis

Sample sizes are indicated in the legends and correspond to biological replicates. Power analysis was performed to estimate the number of animals (two groups, *t* test, G*Power v3.1) with individual estimated experimental effect size, alpha = 0.05, and 95% power. Figure legends include sample size (*n*) information for each treatment group. EV characterization was analyzed with a two-tailed unpaired *t*-test. Biological function terms were reduced by redundancy utilizing REVIGO, reduce+visualize Gene Ontology (v 1.8.1). Unless otherwise stated, statistical analysis and representation was visualized in GraphPad Prism (v 10.0.3).

## Data Availability

The data that support the findings of this study were deposited to NCBI Gene Expression Omnibus (GEO) database and will be publicly available after the manuscript is accepted. GEO submission number (GSE296704 and GSE296705) and reviewer token (38546153).

## Supplementary Materials

The following supporting information can be downloaded at: https://zenodo.org/records/16862195?token=eyJhbGciOiJIUzUxMiJ9.eyJpZCI6ImE3NjVjYjU5LWE2ZWQtNDAzYi04MmU0LTMyMzVkM2Q5MjAxMiIsImRhdGEiOnt9LCJyYW5kb20iOiJlNzg0NjViODMwNTUxOThiMDQ1MzNiNGEyMDY1NGUyNSJ9.5XNIYjRiKllpvlHwNlAXEx7J-uGk1GKNOyQ9xBcmK4DRxa1FLDuDjz5SRA1owsI-iXHjub-mxOR6YgypuBkedA.
